# Integrating point-of-care screening for curable sexually transmitted infections with HIV, syphilis and hepatitis B screening in antenatal care services in Zimbabwe: a mixed-methods process evaluation

**DOI:** 10.1136/bmjgh-2025-019820

**Published:** 2025-12-05

**Authors:** Kevin Martin, Constance R S Mackworth-Young, Ethel Dauya, Rangarirayi Nyamwanza, Chido Dziva Chikwari, Maureen Tshuma, Joseph D Tucker, Victoria Simms, Tsitsi Bandason, Francis Ndowa, Leolin Katsidzira, Owen Mugurungi, Anna Machiha, Remco P H Peters, Michael Marks, Katharina Kranzer, Rashida A Ferrand

**Affiliations:** 1 Department of Clinical Research, London School of Hygiene & Tropical Medicine, London, UK; 2 The Health Research Unit Zimbabwe, Biomedical Research and Training Institute, Harare, Zimbabwe; 3 Department of Global Health and Infection, Brighton and Sussex Medical School, Brighton, UK; 4 Department of Global Health and Development, London School of Hygiene & Tropical Medicine, London, UK; 5 Department of Infectious Disease Epidemiology, London School of Hygiene & Tropical Medicine, London, UK; 6 Skin & Genito-Urinary Medicine Clinic, Harare, Harare Province, Zimbabwe; 7 Internal Medicine Unit, Faculty of Medicine and Health Sciences, University of Zimbabwe, Harare, Zimbabwe; 8 AIDS and TB Unit, Ministry of Health and Child Care, Harare, Zimbabwe; 9 Research Unit, Foundation for Professional Development, East London, South Africa; 10 Department of Medical Microbiology, University of Pretoria, Pretoria, South Africa; 11 Division of Medical Microbiology, University of Cape Town, Cape Town, South Africa; 12 Hospital for Tropical Diseases, University College London Hospital, London, UK; 13 Division of Infection and Immunity, University College London, London, UK; 14 Institute of Infectious Diseases and Tropical Medicine, LMU University Hospital, LMU Munich, Munich, Germany; 15 German Center for Infection Research (DZIF), partner site Munich, Munich, Germany

**Keywords:** Screening, Zimbabwe, Global Health, Health services research, Maternal health

## Abstract

**Introduction:**

Sexually transmitted infections (STIs) in pregnancy are associated with adverse birth outcomes. We investigated the uptake and yield of point-of-care screening for *Chlamydia trachomatis*, *Neisseria gonorrhoeae* and *Trichomonas vaginalis*, integrated with HIV, syphilis and hepatitis B virus (HBV) screening in antenatal care (ANC) in Zimbabwe, and conducted a mixed-methods process evaluation of the strategy.

**Methods:**

A prospective interventional study was conducted in two public-sector ANC clinics in Harare. Clients attending for ANC were screened in parallel for *C. trachomatis*, *N. gonorrhoeae*, *T. vaginalis*, HBV, HIV and syphilis, using four different point-of-care tests. Uptake of STI testing and treatment was recorded. Interviews and focus group discussions with pregnant women, healthcare professionals and the intervention team were conducted and analysed thematically. Implementation, mechanisms of impact and context were explored using the Medical Research Council Process Evaluation Framework.

**Results:**

Between 12 January 2023 and 23 October 2023, there were 13 500 ANC attendances over 207 implementation days. Of 1105 (8.2%) assessed for eligibility, 1103 (99.8%) were eligible, of whom uptake of the full screening package was 91.0% (1004/1103). Curable STI prevalence was 30.7% (308/1003), of whom 303 (98.4%) received same-day treatment. HBV prevalence was 1.4% (14/1003). The prevalence of HIV was 10.5% (105/1003), with 20 (19.0%) being new diagnoses.

Although the intervention was highly acceptable, diagnostic capacity and workload were barriers to recruitment. In particular, the collection and testing of multiple sample types with different tests, with a range of reading times, was challenging.

**Conclusion:**

We demonstrated high levels of acceptability, screening uptake and same-day treatment. However, a low proportion of ANC attendees were enrolled overall, and current technological limitations preclude this particular testing strategy from being scaled up beyond low-volume settings. We recommend investment into the development of STI point-of-care tests with shorter analytic times and research into alternative strategies involving laboratory-based high-throughput testing.

**Trial registration number:**

ClinicalTrials.gov NCT05541081; registered 15 September 2022.

**WHAT IS ALREADY KNOWN ON THIS TOPIC**

- Studies to date have demonstrated that integrated point-of-care screening for sexually transmitted infections in antenatal care is acceptable, with high levels of uptake; however, qualitative and mixed-methods approaches are lacking, which are key to understanding potential scalability.

**WHAT THIS STUDY ADDS**

- We demonstrate how barriers including current technological limitations are amplified by the context of implementation within an overburdened healthcare system, with high workload, unreliable water and electricity supply and high staff turnover.

**HOW THIS STUDY MIGHT AFFECT RESEARCH, PRACTICE OR POLICY**

- This study contributes to gaps in the literature by providing granular-level implementation data, which can be used to guide future evaluations of screening for sexually transmitted infections across different settings.

## Introduction

 Globally, the incidence of curable sexually transmitted infections (STIs) remains high, with funding, advocacy and control lagging behind that for HIV.^1^ In pregnancy, STIs are associated with adverse birth outcomes, including low birth weight, prematurity and stillbirth.^1^,^4^ HIV and syphilis screening in pregnancy is recommended in high-burden resource-constrained settings. More recently, the WHO’s triple elimination initiative encourages countries to commit to the elimination of vertical transmission of HIV, syphilis and hepatitis B virus (HBV); central to the strategy is screening in antenatal care (ANC).^1 5^ However, screening for curable STIs such as *Chlamydia trachomatis*, *Neisseria gonorrhoeae* and *Trichomonas vaginalis* has not been implemented.^1 6^

We hypothesised that screening for curable STIs that builds on existing antenatal HIV/syphilis screening could enhance feasibility, as such screening is already embedded within health services, and ANC is a key point of engagement with health services. The availability of tests that can be used at the point of care also allows for the provision of same-day testing and treatment, potentially reducing the risk of individuals being lost to follow-up.^7^ This study provides an opportunity to additionally evaluate integration of HBV screening, which is not yet implemented in Zimbabwe, prior to its introduction.^8^ While ANC screening for curable STIs has been shown to be broadly acceptable and feasible, prior studies have focused on uptake of screening and treatment.^9^,^13^ At present, there is a lack of a mixed-methods framework or theory-driven process evaluation for such interventions, to understand in depth the facilitators and barriers to implementation and scale-up.^11^

The overall aim was to assess a comprehensive point-of-care STI testing strategy in antenatal settings in Harare, Zimbabwe, using a mixed-methods process evaluation framework, with a focus on implementation, mechanisms of impact and context. 

## Methods

### Study design and setting

A prospective interventional study was conducted in two urban primary healthcare clinics (PHCs) that serve high-density lower-income communities in southwest Harare, Zimbabwe. PHCs provide nurse-led ANC and routine delivery services, with referral to hospital available for complications. Opt-out HIV and syphilis testing are provided as standard of care and syndromic management used for other STIs.^14^ At the time of the study, HBV screening was not standard of care, but implementation was being planned for in line with WHO recommendations.^1^ Site A has 3 days allocated for ANC bookings (initial registration and assessment of pregnant women) per week, 1 day for follow-ups and 1 day for postnatal visits. Site B has 2 days allocated for bookings, 2 days for follow-ups and one postnatal day. Recruitment took place on booking and follow-up days. In each clinic, there was a dedicated ANC building with a central waiting room, off which were other rooms or sectioned areas for different ANC activities. At both sites, the intervention team had a dedicated room in the ANC building for screening, either off an adjacent corridor from the waiting room (site A) or directly off the waiting room (site B). All intervention-specific activities took place in the study room. Both were high-volume sites, with 4565 and 2486 ANC bookings at sites A and B in 2022, respectively.^15^

### Study population

Any pregnant woman attending one of the study clinics for ANC was eligible for participation, regardless of age, stage of pregnancy or antenatal visit type. Exclusion criteria were prior enrolment into the study or being unable to provide informed consent. Reasons for exclusion and for declining participation were documented.

We had provisionally estimated that eight participants could be recruited per day. However, on implementation, daily recruitment was limited to five participants due to logistic feasibility. Prior to enrolment, the study team provided some information on the study to all ANC clients in the waiting room. Subsequently, every fifth individual seated sequentially in the main ANC waiting area (up to five individuals) was invited to attend an indepth information session, where the study team talked through the study and consent form in detail and were invited to participate. If any of the selected individuals declined participation, this was recorded and another individual in the same sequence as previously was invited to participate. If there were not enough attendees to allow for every fifth individual to be invited, then a sequence with a lower denominator (eg, every third individual) was used.

### Intervention procedures

Detailed study procedures are described in the previously published protocol.^16^ A schematic diagram in online supplemental material A also details the order of daily study activities. The study team consisted of one nurse and one research assistant at each PHC. Participants were enrolled between 12 January 2023 and 23 October 2023. Following written informed consent, two blind vaginal swabs were collected by the intervention team nurse for testing for *T. vaginalis* using the OSOM *Trichomonas* Rapid Test (Sekisui) and for testing for *C. trachomatis*/*N. gonorrhoeae* using the Xpert CT/NG (Cepheid). A dedicated four-module GeneXpert machine was situated in the clinic room at each site, with a back-up powerpack (Goal Zero Yeti 500X) used as an uninterruptible power supply and back-up power source when mains power was unavailable. If additional consent was provided, three additional vaginal swabs were collected for other diagnostic evaluation studies.

Data were collected from each participant using an interviewer-administered questionnaire using Open Data Kit on tablet computers. Data collected included sociodemographic information, pregnancy history, sexual behaviours and current clinical symptoms. Women were tested by our study team for HIV and syphilis with the SD Bioline HIV/syphilis Duo (Abbott Laboratories) using a fingerprick blood sample, as per standard of care in Zimbabwe. Screening of women for HBV was performed on the same sample using the Determine HBsAg2 (Abbott Laboratories) rapid test kits. Only treponemal antibody tests were used for syphilis, and so, we were unable to distinguish between current and past infection. Samples were taken from participants before the questionnaire was administered so that sample processing could proceed during data collection. When not involved in study procedures, participants attended routine ANC activities, such as blood pressure checks, height, weight, physical examination and gestation period calculations, tetanus vaccination, routine blood sampling including full blood count and health education.

Once all test results were available, each participant returned for post-test counselling and treatment if required. Partner notification slips were provided to participants with a positive STI test or who were treated for an STI syndrome. Due to the potential for negative consequences of partner notification, such as relationship breakdown or intimate partner violence,^17^ we aimed to support participants to come to the decision that was right for them, whether that was informing or not informing their partner. Participants with vaginal discharge syndrome were given the option of receiving full treatment immediately or receiving tailored treatment based on aetiological results. Treatment was provided to partners free of charge if they returned to the study team for treatment. For pregnant women, treatments for *C. trachomatis* (oral azithromycin) and *N. gonorrhoeae* (intramuscular ceftriaxone) were single dose therapy provided on the same day, whereas for *T. vaginalis* a 7-day oral course of metronidazole was provided. Participants diagnosed with HBV were referred to a specialist hepatology clinic appointment, and birth dose vaccination was arranged for their newborn. Universal HBV birth dose vaccination is not currently standard of care in Zimbabwe. Participants newly diagnosed with HIV were referred for onward care and partner notification as per existing clinic processes.

### Process evaluation framework

A concurrent parallel mixed-methods process evaluation was conducted, guided by the Medical Research Council (MRC) (UK) Process Evaluation Framework.^18^ Quantitative and qualitative data were similarly weighted and collected and analysed in parallel prior to comparison and integration.^19^ This allowed for triangulation of data to comprehensively address research questions. The focus was on understanding what was implemented and how; how the intervention led to change and how context affected implementation and shaped outcomes. Steckler and Linnan’s process evaluation framework also guided the choice of specific research domains related to implementation, particularly the focus on fidelity and reach/coverage.^20^ The process evaluation domains, questions and indicators are shown in online supplemental table B. The original logic model describing the proposed theory of change is included in the published protocol.^16^

### Data collection

Quantitative data collected included uptake of testing, treatment and partner notification. The number of clients attending for ANC was extracted from the clinic register. Qualitative data included semistructured interviews, focus group discussions, structured, unstructured and time-in-motion observations, field notes and context diary entries. Interviews and discussions were guided by topic guides. Participants for interviews and group discussions were purposively selected to ensure a range of demographics and test results for ANC clients and role for healthcare workers. This process involved regularly reviewing such data from previously enrolled participants to identify groups who were underrepresented in the data at that point.

A total of 57 interviews were conducted with pregnant women (n=25), partners (n=10), HIV testing counsellors (n=7), midwives (n=6), nurses-in-charge (n=2), a specialist hepatology physician (n=1) and the nurses and research assistants delivering the intervention (n=4, with two members being interviewed twice, midway and post implementation). Interviews were conducted in person by a female interviewer trained in qualitative interviewing and the particular topic guide, who was not involved in service delivery (RN), in Shona and/or English. One interview with a specialist physician was conducted by the study principal investigator (KM, male), in English. The interviews lasted a median of 26:59 min (range 8:35–85:29). The broad range in interview duration is a result of interviews with different groups of interviewees using different topic guides.

Two focus group discussions with pregnant women were conducted, one with 11 participants (duration: 72:53 min) and one with five participants (duration: 58:19 min). Focus group discussions were conducted in Shona and led by RN and MT together, with KM observing. All interviews and focus group discussions were audio-recorded and transcribed directly into English by RN. Time-in-motion studies were conducted on 12 study days, where RN observed in the study PHC and documented the start and end time for each study activity for individual participants.

### Data analysis

STATA V.18.0 (StataCorp, Texas, USA) was used for quantitative data analysis. The target sample size was 1000 pregnant women to be enrolled and screened.^16^ The primary quantitative outcomes were the composite and individual prevalence of *C. trachomatis*, *N. gonorrhoeae*, *T. vaginalis*, syphilis and HBV. STI prevalence was calculated as the number of participants with a positive STI result divided by the total number of individuals with test results. STI yield was calculated as the number of participants with a positive STI result divided by the total number of individuals assessed as being eligible. Descriptive statistics were used to demonstrate uptake along the STI care cascade.

For qualitative data, NVivo V.14 (QSR International) was used to assist with coding transcripts. Data were analysed thematically and deductively, within each of the three main process evaluation constructs, namely: what was implemented and how; how the intervention led to change and how context affected implementation and shaped outcomes.^21^ Although topic guides were designed to align with specific research questions and domains, open coding was used, and, within each construct, codes were generated and grouped into themes inductively. Following familiarisation with the data, coding and initial theme development by KM, themes were reviewed and refined by KM, CRSMY and RAF. The interaction of the themes and subthemes with the process evaluation domains and the pathway to impact is shown in online supplemental figure C. Themes, subthemes and supporting quotes are reported in online supplemental table D. Partner notification outcomes are briefly presented here as they are the focus of another manuscript.^22^ Data have been reported according to the Standards for Reporting Implementation Studies and the Standards for Reporting Qualitative Research.^23 24^

### Patient and public involvement

Antenatal clients and their partners were interviewed before and during implementation, as part of the process evaluation. This was a dynamic process, with adaptations to screening made based on this input on an ongoing basis. Patients and the public were not involved in initial study design.

### Reflexivity

A structured reflexivity statement can be found in online supplemental material E.

## Results

A summary of the process evaluation findings by domain is shown in online supplemental table B. The interaction of the themes and subthemes with the process evaluation domains and the pathway to impact is shown in online supplemental figure C.

### Implementation

#### Uptake and yield of STI screening

**Figure 1:**
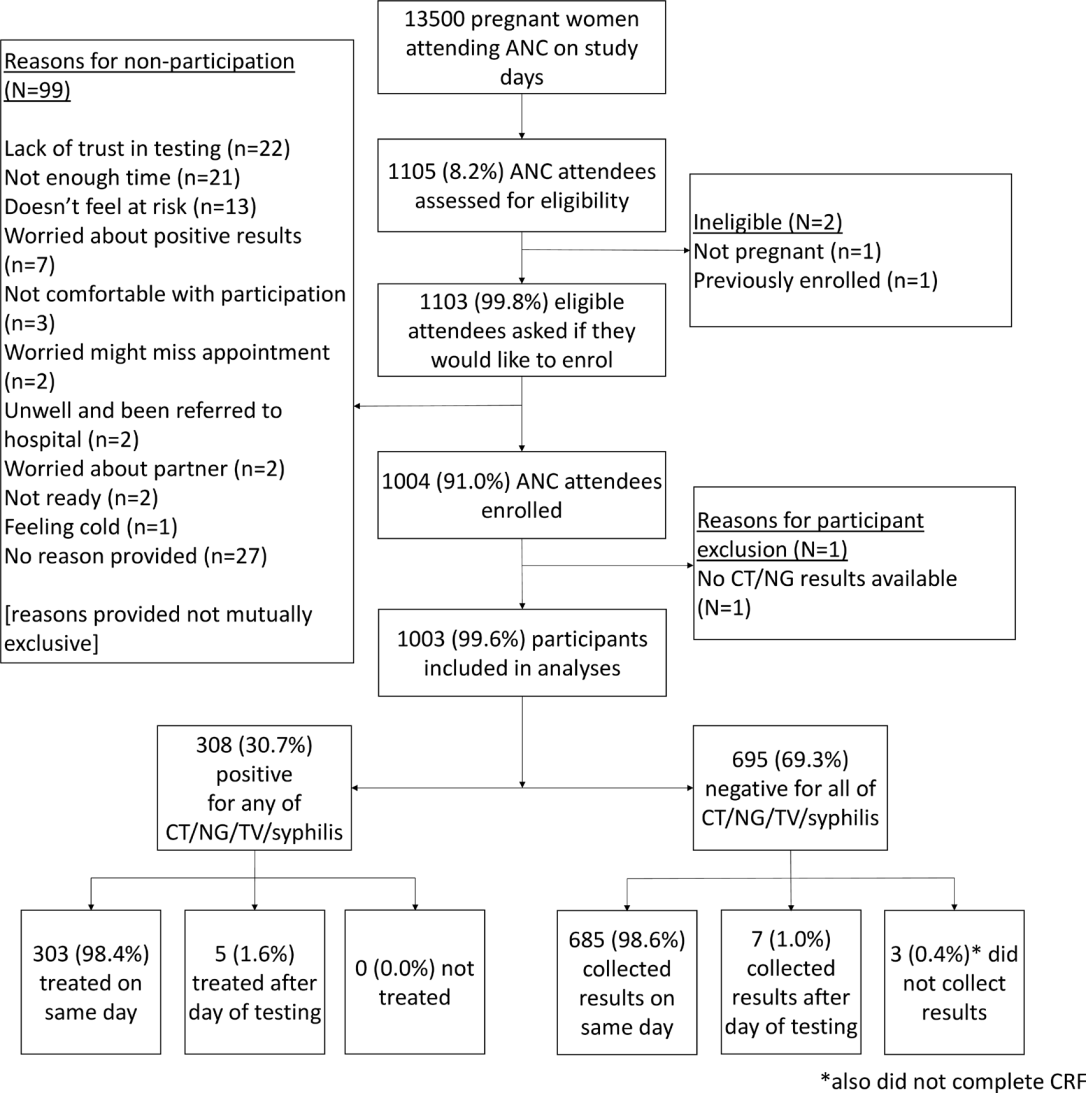
Testing cascade for curable sexually transmitted infections. ANC, antenatal care; CRF, case report form; CT*, Chlamydia trachomatis*; NG*, Neisseria gonorrhoeae*; TV, *Trichomonas vaginalis*.

There were 13 500 ANC attendances recorded across two clinics over 207 implementation days; 1105 women (8.2%) were assessed for eligibility, of whom 1103 (99.8%) were eligible (figure 1). Table 1 shows the prevalence of individuals assessed for eligibility, as a proportion of the number of antenatal attendees, disaggregated by site and by type of ANC day. Overall enrolment and uptake of the full screening package was 91.0% (1004/1103). All 1004 participants accepted all available tests, with 1003 having a full set of STI results. Participant characteristics (n=1000) are shown in table 2. Three participants did not return to complete the questionnaire. The median age of participants who enrolled was 25 years for both those who enrolled (IQR 21–31 years) and did not enrol (IQR 22–29 years). Median reported gestational age for enrolled participants was 28 weeks (IQR 23–33, n=895).

**Table 1:** Prevalence of individuals assessed for eligibility, as a proportion of the number of antenatal attendees, disaggregated by site and by type of ANC day

ANC day	Site A	Site B	Overall
Booking	14.7% (533/3629)	29.0% (174/599)	16.7% (707/4228)
Follow-up	2.5% (192/7787)	13.3% (197/1476)	4.2% (389/9263)
Other*	100.0% (9/9)	–	100.0% (9/9)
Overall	6.4% (734/11425)	17.9% (371/2075)	8.2% (1105/13500)

* Nine pregnant women were opportunistically screened and enrolled after they inadvertently attended on a postnatal day rather than a booking day, but clinic staff still completed their booking processes.

ANC, antenatal care.

**Table 2:** Characteristics of participants recruited into the study (n=1000 unless otherwise stated)*

Variable	N (%)
Sociodemographic	
Clinic site	
Site A	701 (70.1%)
Site B	299 (29.9%)
Age (years)	
15 – 19	134 (13.4%)
20 – 24	330 (33.0%)
25 – 29	255 (25.5%)
30 – 34	158 (15.8%)
35+	123 (12.3%)
Education level	
No formal education	3 (0.3%)
Primary	82 (8.2%)
Secondary	865 (86.5%)
Vocational	11 (1.1%)
Higher/University	39 (3.9%)
Current employment status	
Unemployed	518 (51.8%)
Student	14 (1.4%)
Self-employed/business owner	119 (11.9%)
Salaried employment	73 (7.3%)
Informal work	276 (27.6%)
Average monthly household income	
US$0 – 50	59 (5.9%)
US$51 – 100	154 (15.4%)
US$101 – 200	241 (24.1%)
US$201 – 500	289 (28.9%)
US$501 – 1000	47 (4.7%)
US$>1000	14 (1.4%)
Don’t know	196 (19.6%)
Relationships and pregnancy	
Relationship with father of child	
Married	551 (55.1%)
Not married, but living together	360 (36.0%)
Not married or living together	54 (5.4%)
No relationship	35 (3.5%)
Pregnancy planning (N = 970)	
Wanted to become pregnant	570 (58.8%)
Would have preferred to put it off a while	272 (28.0%)
Did not want to become pregnant	128 (13.2%)
Number of previous pregnancies	
0	284 (28.4%)
1	264 (26.4%)
2	222 (22.2%)
3+	230 (23.0%)
Gestational age at enrolment (weeks) (N = 895)*	
2 – 12	18 (2.0%)
13 – 27	386 (43.1%)
28 - 41	491 (54.9%)
Sexual history	
Perceived STI risk	
No risk	506 (50.6%)
Unlikely	29 (2.9%)
Likely or very likely	228 (22.8%)
Unsure	237 (23.7%)
Number of partners in past 30 days	
0	102 (10.2%)
1	895 (89.5%)
2	3 (0.3%)
Condom use during vaginal sex	
Never or rarely	885 (88.5%)
Sometimes	98 (9.8%)
Most of the times	6 (0.6%)
Always	11 (1.1%)
Suspicion of partner(s) having other sexual partners currently (N = 968)	
Yes	399 (41.3%)
No	325 (33.6%)
Don’t know	243 (25.1%)
Received or told they would receive gifts, financial support, or other material support in exchange for sex in last 12 months	
Yes	35 (3.5%)
No	962 (96.2%)
Don’t know	3 (0.3%)

* Gestational age at enrolment was extracted from antenatal records on day of attendance. These data were missing for 105 participants due to challenges in calculating gestational age, with recommendations for an ultrasound scan to assist in calculations.

STI, sexually transmitted infection.

The prevalence of individual STIs was: *C. trachomatis* 18.5% (186/1003, 95% CI 16.2 to 21.1%); *N. gonorrhoeae* 4.0% (40/1003, 95% CI 2.9 to 5.4%); *T. vaginalis* 12.4% (124/1003, 95% CI 10.4 to 14.6%); syphilis 3.5% (35/1003, 95% CI 2.4 to 4.8%) and HBV 1.4% (14/1003, 95% CI 0.8 to 2.3%). The prevalence and yield of the intervention for at least one non-HIV infection (*C. trachomatis*, *N. gonorrhoeae*, *T. vaginalis*, syphilis or HBV) was 31.5% (316/1003, 95% CI 28.7 to 34.5%) and 28.6% (316/1103, 95% CI 26.0 to 31.4%), respectively. The prevalence of HIV was 10.5% (105/1003, 95% CI 8.5 to 12.5%), with 20 (19.0%) being new diagnoses. The prevalence of HIV-HBV co-infection was 0.2% (2/1003, 95% CI 0.02 to 0.7%).

### Fidelity

Fidelity of the intervention focused on the provision of same-day testing and treatment. The intervention was conducted by a dedicated intervention team with enrolment of a maximum number of five participants per day in order to ensure adherence to the protocol and to enable collection of additional data. As a result, high levels of fidelity were possible, as the team was not overburdened by the numbers needed to screen or by the limitations of the health system, with 99.7% (1000/1003) of participants collecting their results, 98.5% (988/1003) on the same day. Of 308 (30.7%) participants with a curable STI (*C. trachomatis*, *N. gonorrhoeae*, *T. vaginalis* or syphilis), 303 (98.4%) received same-day treatment, with the remaining five participants treated on a later date. Off-site testing for *C. trachomatis* and *N. gonorrhoeae* at our laboratory was required for 0.7% (7/1003) of participants, due to a technical problem with GeneXpert laptop keyboard (n=5), the GeneXpert laptop battery running out (n=1) and a GeneXpert error late in the day (n=1). For the 988 participants who received same-day results, the vast majority (97.4%; 962/988) stated that they did not have to wait beyond the duration of the ANC visit to receive their result. Additional outputs related to fidelity are presented in online supplemental table B, including the additional time spent at clinic for those who did have to wait longer, number of days screening was offered at clinic, hepatology referral and HBV birth dose vaccine outcomes and site-specific alterations to implementation at each clinic.

Despite the controlled conditions, challenges with high likelihood of disruption when implemented as routine were identified, particularly related to the heavy reliance on technology in a setting where maintenance and repairs were often subject to significant delays. For example, a malfunctioning laptop keyboard could have significantly derailed screening. Keyboard replacement took several months, during which time a spare laptop with GeneXpert software installed was made available. The capacity of the 4-module GeneXpert machine was considered a limitation to scalability, with one intervention team member suggesting that laboratory testing would be required to scale up screening. Overall, GeneXpert errors affected 25 participants. In all but one, a sample was re-run, and an actionable result was obtained. For the remaining individual, there was insufficient volume left in the swab container to re-run the sample, and the woman did not want to provide another swab.

### Mechanisms of impact

#### Responses to and interactions with the intervention

Overall, the intervention had high levels of acceptability to clients. Alongside a high rate of enrolment, all enrolled participants underwent testing for all six STIs. The most common reasons for declining participation were a lack of trust in testing (n=22); a lack of time (n=21); not feeling at risk of STIs (n=13); and worries about positive results (n=3) (figure 1).

Many participants reported feeling ‘*lucky*’ to have been enrolled. One aspect that was noted to enhance acceptability was the curability of the majority of infections screened for, which were perceived as much less serious than HIV, which was considered by some to be their *‘worst fear’*. One participant reported that they *‘didn’t really feel moved by the results because the nurse told me that it is treatable*’. There were also many requests for the intervention to test more than five clients per day and to test beyond pregnant women. The intervention screening component was reported by participants to be beneficial in order to detect asymptomatic infection, catch infections early, assess partner fidelity, know their status and protect their baby. Many participants also reported appreciation of the results being available on the same day and of the accompanying education, counselling and support provided by the intervention team.

Provider collection of vaginal swabs was largely reported to be very acceptable, although a few participants reported feeling shy. Participants often reported that they had an expectation of pain or discomfort that was not realised. They also appreciated distraction techniques used by the intervention team.

For participants diagnosed with HBV, qualitative data suggested relatively high levels of acceptability for both the appointment with a hepatologist and birth dose vaccination; however, uptake of both was more modest (supplementary table B). One participant initially reported they were worried as, *‘when you hear that you are being transferred there, you start wondering like what kind of condition is hepatitis B such that a specialist has to be consulted’.* However, specialist input and counselling generally appeared to reassure clients. One aspect of care that was suboptimal was HBV contact tracing, where no contacts came forward for testing. In at least one instance, the male partner of an index case prevented testing of both himself and their other children.

### Interactions and consequences

The interactions and consequences of the intervention focused on ‘integration’ of the intervention with routine ANC. As a result of having a dedicated team and the requirements of a research study, some aspects of implementation and evaluation do not reflect ‘real world’ settings. Challenges to integration included a high staff turnover and a heavy reliance on locum staff at clinic A, such that new midwives on duty were sometimes unaware of the study. There were also a few reports that intervention processes disrupted routine ANC processes. Procedures performed as part of the research, including informed consent and questionnaires, meant that the intervention team had to balance time dedicated to research with the time spent on delivering the intervention. Table 3 details average time spent on individual study activities. While more women could have been screened if implemented as routine care, the number of staff and the testing capacity are unlikely to have been sufficient to offer screening to all women attending ANC.

**Table 3:** Time-in-motion studies showing average time spent on intervention activities at sites A and B and overall, based on 12 study days (site A booking=4 days; site A follow-up=1 day; site B booking=5 days; site B follow-up=2 days)

Activity	Site A average (hours/min)	Site B average (hours/min)	Overall average (hours/min)
Setting up/waiting times for clients	00:32	00:38	00:36
Group consent	00:38	00:25	00:30
Individual consent (×5)	00:24	00:20	00:21
Swab collection (×5)	00:13	00:14	00:14
Fingerprick testing (×5)	00:09	00:10	00:09
Study questionnaire (×5)	01:38	00:53	01:12
Results, counselling andf treatment (×5)	00:17	00:13	00:15
Updating clinical forms	00:35	00:28	00:31

### Context and implications for real world implementation

#### Socioeconomic

The broader economic environment within Zimbabwe is unstable. Alongside infrastructural challenges, such as irregular water, sanitation and electricity supply, a lack of local employment also contributes to familial disruption as individuals have to leave the country or travel from rural to urban settings for work. High levels of impoverishment also affect access to healthcare. Many participants reported difficulties raising the booking fee covering all ANC activities, which was US$25 at both clinics. The availability of donor-funded results-based financing vouchers, administered through the Ministry of Health and Child Care, facilitated access to ANC for those unable to afford clinic fees. However, sometimes women were told that *‘their voucher is not appearing in the system*’. This reportedly led to late booking, with some pregnant mothers only attending *‘when they are due already, like 39 weeks*’. The majority (601/999, 60.2%) also reported that they incurred transport costs to/from the clinic. Crucially, the package of testing in this intervention was provided for free, but many participants stated that if they had to pay for such tests, they either would not be able to or would need time to raise the funds. Similarly, a participant diagnosed with HBV missed a hepatology appointment as she was unable to raise the funds for transport (which would have been reimbursed on attendance). Concerns were raised by the specialist hepatologist about the impact this will have on retention in care for these participants. This also demonstrates how the provision of same-day results and treatment was important as returning to clinic would have been challenging for some clients.

Social issues reported in local communities included drug and alcohol abuse, sex work and high-risk sexual behaviours. There was also evidence of adolescent pregnancies and relationships with age gaps and in-built power imbalances, demonstrating the vulnerability of youth. In one instance, after a 15-year-old became pregnant, her partner disappeared and the parents reportedly *‘chased away*’ their daughter. Social isolation and a lack of support networks also came up repeatedly, with several saying that they *‘don’t interact with anyone*’ in their community. As a result, the intervention team often had to take on additional supportive and counselling roles for participants facing challenging situations.

#### Cultural

There were several cultural-level themes related to stigma, religious beliefs and tradition and patriarchy and gender roles. Participants discussed a lot of stigma surrounding STIs and HIV, with participants reporting that people do not talk about STIs or want others to know they have them, or using terminology such as being *‘rotten already with HIV or other STIs’*. People felt that there was a strong association between HIV and STIs and ‘promiscuity’, with both pregnant women and staff reporting that STIs were acquired through *‘having multiple sexual partners’*. These stigmatising attitudes contributed to women finding it difficult to inform partners about STIs, with only one-third of partners being treated, thereby risking re-infection from untreated partners (supplementary table B). Stigma also influenced how the intervention was perceived, with some not wanting to have a dedicated *‘STI testing*’ room that was visible to others, as they felt *‘embarrassed to be seen getting up*’ for testing, and preferred a room *‘away from the ANC building’.* The stigma associated with HIV and STIs was felt to also have ramifications for HBV. The specialist hepatologist noted that, were HIV and HBV screening to be integrated, there was a risk that HBV would be stigmatised by association.

Patriarchal norms were very evident; relationship decisions were often male decisions, for example on condom use. Similarly, within the intervention, HBV birth dose vaccination and contact tracing were not always possible if vetoed by the participant’s partner. There was an acceptance that men would be *‘cheating*’ but there was *‘nothing that I can do’*. Women reported wanting STI testing as they did not trust their husbands, enhancing intervention acceptability. Furthermore, the onus was placed on women to prevent their partners cheating by ensuring they get their *‘full conjugal rights from conception till they give birth*’.

#### Health system-level

The intervention was implemented within a healthcare system subject to many competing priorities beyond its capacity, including a cholera outbreak. This manifested itself in poor basic clinic infrastructure and stockouts of test kits, medicines and other basic commodities. As a result, despite already paying ANC access fees, clients incurred further out-of-pocket expenditure or went without medication. Additional fees would be required for ultrasound scans or if an ambulance was required. As a result of heavy staff attrition, attributed to low salaries and a difficult working environment, there were staff shortages and a reliance on locum staff, which made integration of the intervention with routine care more challenging.

Of note, even with a dedicated team enrolling a maximum of five participants per day, the intervention team reported that the workload was a challenge. Implementation within busy antenatal departments, and navigating different processes to ensure testing and treatment was complete and keeping track of participants was reported to be ‘*hectic’*. Furthermore, for each client, the use of multiple different samples and tests, all of which had different reading times, made conducting testing very intense, raising questions about how implementation would be possible without a dedicated team or multiplexing real point of care tests.

### Clinic-level

Finally, implementation was affected by several clinic-level factors. Erratic water supply led to disruption as handwashing took additional time, and sometimes the clinic would be closed if there was a persistent absence. For telephone follow-up, mobile network at and around site A was particularly challenging and led to difficulties in following up participants and gathering post-natal data. Electricity supply at both clinics was unreliable. The GeneXpert machine was powered through a power pack to prevent disruption to screening. Space was another consideration. In each site, there was access to a single room with limited options for additional private space—this led to inefficiencies as the nurse and research assistant could not attend to two different clients concurrently. Finally, the GeneXpert machine, laptop and power pack were transported daily to the clinic from the office, on the advice of clinic staff, as overnight security was not guaranteed.

## Discussion

We found a high prevalence of STIs among pregnant women attending for ANC in Zimbabwe. Incorporating additional tests for *C. trachomatis*, *N. gonorrhoeae*, *T. vaginalis* and HBV into ANC, alongside routine HIV and syphilis screening, was highly acceptable to pregnant women. This high acceptability assumes the women are able to access ANC, the tests are free of charge and there is an understanding that they are beneficial for themselves and their babies. While the intervention had high uptake among those who were offered it, it had low coverage, reaching only a small proportion of total antenatal attendees. This has implications for the scalability of the intervention; given the very high number of pregnant women booked into ANC, testing capacity is central to the feasibility of implementation at scale.

The high prevalence of STIs is consistent with other studies among pregnant women in Southern Africa^25^,^27^ and is indicative of broader regional and global challenges to STI control. The high uptake of STI screening within an integrated package observed in our study concurs with other settings.^9 12 13 28^ This high uptake reflects the high level of acceptability of screening, which we heard from participants was due to an appreciation of being made aware of asymptomatic infections, allowing them to protect themselves and their baby and receiving same-day results and treatment. Partner treatment was challenging, with only one in three partners attending for treatment, and is discussed further in another manuscript.^22^

In this study, 98.4% of participants with a curable STI received same-day treatment. Other studies using the GeneXpert for point-of-care STI testing in ANC in resource-limited settings also reported between 80.0% and 98.6% participants screened receiving same-day treatment,^9 12 13 29^ demonstrating that same-day testing and treatment is possible using such a strategy. In this study, same-day treatment provision was enabled by limiting the number of women screened to five a day due to both the capacity of the GeneXpert machine and lengthy research processes (an average of 2 hours was spent on consenting and study questionnaires each day, for five participants). This meant that on booking days, we were only able to approach 16.7% of antenatal attendees. This was lower at site A (14.7%), which is a very high throughput clinic, compared with site B (29.0%). In other studies, 56% and 83% of ANC attendees were enrolled in Papua New Guinea and Botswana, respectively.^9 13^ Based on implementation experiences in our study, it is unlikely that a model with the current resources available (ie, one four-module GeneXpert machine) would be suitable for implementation at a clinic like site A. However, it may still be an appropriate model for low-volume clinics booking smaller numbers of clients per day. Similar findings were also reported in non-ANC settings in Zimbabwe. An evaluation of on-site *C. trachomatis*/*N. gonorrhoeae* screening using GeneXpert for youth in urban community settings in Zimbabwe found significant issues with testing capacity and flow, resulting in an inability to reliably provide same-day results and treatment to all those screened.^30^

The HBV prevalence was 1.4% (1.3% in HIV negative individuals), which is similar to the prevalence of 1.2% among HIV-negative pregnant women in Harare reported in a 2023 study.^31^ We referred clients diagnosed with HBV for specialist hepatology review within secondary care, with clinic fees and transport costs covered by the study, of whom 71.4% attended. This suggests that, even with the removal of financial barriers, challenges remain to engage clients in ongoing care. Additionally, if universal HBV screening is implemented as part of routine practice, consideration must be made for how those diagnosed will be managed, as numbers diagnosed would likely outstrip the current capacity of public sector hepatologists in Zimbabwe. Consideration would need to be given to the development and implementation of algorithms and guidelines to allow for HBV to be managed outside of specialist settings.^32^ The four newborns not receiving the birth dose vaccination were either due to a lack of consent from one of the parents or administrative/logistics issues. At least some of these instances of non-vaccination may have been averted had birth dose vaccination been universally implemented in Zimbabwe, as recommended by the WHO.^1^ In 2021, Dzingirai *et al* reported that challenges to implementation of birth dose vaccination in Zimbabwe included a lack of funding, cold chain facilities and difficulties reaching infants within the first 24 hours of birth to administer the vaccine.^8^ Birth dose vaccination is an essential component for the elimination of vertical transmission of HBV; as a result, identification of the current barriers to a universal rollout and strategies to resolve them is imperative.^1^

Other important aspects related to the implementation of screening that also impact scalability included workload, reliance on technology and integration with existing ANC processes. One particular aspect was the use of multiple different testing platforms, each with different reading or sample processing times, necessitating multiple timers so that the team knew when to read results. Given the overall challenges reported in this study when screening even a limited number of attendees on site, multiplex testing or a hub-and-spoke model with testing in a laboratory may be more appropriate. Such challenges are likely to be amplified if sample collection and screening is being conducted by routine ANC staff in settings with high staff turnover and where quality control is difficult to implement (i.e outside of a laboratory). The reliance on the GeneXpert machine itself was also challenging for the intervention team. Errors on the GeneXpert necessitated re-running of samples, leading to disruption to usual processes, which has previously been reported in community settings in Zimbabwe.^30^ They also had to conduct such sample processing alongside sample collection, counselling and provision of treatment, while keeping track of participants and trying to integrate with existing ANC processes. Consideration should therefore be given to the GeneXpert device or other diagnostic being laboratory-based, with a laboratory technician responsible for sample processing.

The context was key to understanding the challenges to implementation of STI screening in ANC. At a clinic level, water and electricity were not reliable; similar conditions were reported in a study conducted in Botswana, where a lack of running water or electricity temporarily halted enrolment.^13^ To address this, we used a back-up power pack and brought our own water supply, which worked well. Additionally, the healthcare system is suffering from years of underinvestment with infrastructure in a poor state and out-of-pocket expenditure required for ANC (if not eligible for a voucher) and other essentials, such as antibiotics. Staffing was challenging and there was a heavy reliance on locum staff. Furthermore, it was clear that many clients would have been unable or unwilling to pay additional costs for such tests if required. We navigated these difficulties through having a dedicated intervention team and by providing these additional services to clients for free. Both staffing and funding are important considerations for scalability. Without dedicated staff, implementation at scale would be challenging. Additionally, given the precarious funding situation of the Zimbabwean healthcare system and already heavy reliance on out-of-pocket expenditure, additional costs for STI screening are unlikely to be affordable within the national health budget envelope, and alternative funding mechanisms would likely be required.^33^

The strengths of this study are its relatively large sample size and the mixed-methods study design within the MRC process evaluation framework, which allowed us to robustly assess this testing strategy within this context. The main limitation was that testing was not wholly representative of how testing would integrate into existing government clinical services. It is likely that without additional staff, the strategy would not be feasible to deliver in its current form within routine services. The results are likely generalisable to other urban centres in Southern Africa, and less so to rural settings or those with lower client numbers. We also do not provide an indication of how testing would work with other diagnostic models, for example, if centralised laboratory testing was used, where challenges related to follow-up and treatment of individuals with STIs are likely to be more prevalent.

## Conclusion

We have demonstrated that implementation of a comprehensive antenatal STI screening strategy is highly acceptable, with high levels of uptake and same-day treatment. However, we identified key barriers and bottlenecks which are likely to make implementation at scale, with currently available technologies, highly challenging. Given the limitations associated with both testing throughput and infrastructure requirements using GeneXpert, future implementation designs should consider approaches using off-site processing in a laboratory with high throughput technologies. Furthermore, notwithstanding the difficulties associated with using multiple testing platforms, given the prohibitive costs associated with molecular testing, further work on lateral flow assays for *C. trachomatis* and *N. gonorrhoeae* is required. Evaluations of implementation must also take place fully embedded within public sector health services and outside of the scope of research, to truly understand how implementation would occur in such settings. Finally, given the degree to which the healthcare system in Zimbabwe is already severely overburdened, robust evidence of the clinical effectiveness and cost-effectiveness of such screening must be available to justify the trade-offs required for implementation.

## Supplementary material

10.1136/bmjgh-2025-019820 online supplemental file 1

10.1136/bmjgh-2025-019820 online supplemental file 2

10.1136/bmjgh-2025-019820 online supplemental file 3

10.1136/bmjgh-2025-019820 online supplemental file 4

10.1136/bmjgh-2025-019820 online supplemental file 5

## Data Availability

Data are available in a public, open access repository.

## References

[1] World Health Organization (2022). Global health sector strategies on, respectively, HIV, viral hepatitis and sexually transmitted infections for the period 2022-2030.

[2] Tang W, Mao J, Li KT (2020). Pregnancy and fertility-related adverse outcomes associated with *Chlamydia trachomatis* infection: a global systematic review and meta-analysis. Sex Transm Infect.

[3] Vallely LM, Egli-Gany D, Wand H (2021). Adverse pregnancy and neonatal outcomes associated with *Neisseria gonorrhoeae:* systematic review and meta-analysis. Sex Transm Infect.

[4] Van Gerwen O, Craig‐Kuhn M, Jones A (2021). Trichomoniasis and adverse birth outcomes: a systematic review and meta‐analysis. BJOG.

[5] Cohn J, Owiredu MN, Taylor MM (2021). Eliminating mother-to-child transmission of human immunodeficiency virus, syphilis and hepatitis B in sub-Saharan Africa. Bull World Health Organ.

[6] Medline A, Joseph Davey D, Klausner JD (2017). Lost opportunity to save newborn lives: variable national antenatal screening policies for Neisseria gonorrhoeae and Chlamydia trachomatis. Int J STD AIDS.

[7] Murtagh MM (2019). The Point-of-Care Diagnostic Landscape for Sexually Transmitted Infections (STIs).

[8] Dzingirai B, Katsidzira L, Matyanga CMJ (2021). Progress on the elimination of viral hepatitis in Zimbabwe: A review of the policies, strategies and challenges. J Viral Hepat.

[9] Badman SG, Vallely LM, Toliman P (2016). A novel point-of-care testing strategy for sexually transmitted infections among pregnant women in high-burden settings: results of a feasibility study in Papua New Guinea. BMC Infect Dis.

[10] Gadoth A, Shannon CL, Hoff NA (2020). Prenatal chlamydial, gonococcal, and trichomonal screening in the Democratic Republic of Congo for case detection and management. Int J STD AIDS.

[11] Martin K, Wenlock R, Roper T (2022). Facilitators and barriers to point-of-care testing for sexually transmitted infections in low- and middle-income countries: a scoping review. BMC Infect Dis.

[12] Morikawa E, Mudau M, Olivier D (2018). Acceptability and Feasibility of Integrating Point-of-Care Diagnostic Testing of Sexually Transmitted Infections into a South African Antenatal Care Program for HIV-Infected Pregnant Women. Infect Dis Obstet Gynecol.

[13] Wynn A, Ramogola-Masire D, Gaolebale P (2016). Acceptability and Feasibility of Sexually Transmitted Infection Testing and Treatment among Pregnant Women in Gaborone, Botswana, 2015. Biomed Res Int.

[14] Zimbabwe Ministry of Health and Child Care (2019). National STI management guidelines.

[15] Harare City Health Department (2022). Report on the city health department 2022.

[16] Martin K, Dziva Chikwari C, Dauya E (2023). Investigating point-of-care diagnostics for sexually transmitted infections and antimicrobial resistance in antenatal care in Zimbabwe (IPSAZ): protocol for a mixed-methods study. BMJ Open.

[17] Lariat J, Chikwari CD, Dauya E (2023). *"It’s not safe for me and what would it achieve?"* Acceptability of patient-referral partner notification for sexually transmitted infections to young people, a mixed methods study from Zimbabwe. *Sex Reprod Health Matters*.

[18] Moore GF, Audrey S, Barker M (2015). Process evaluation of complex interventions: Medical Research Council guidance. BMJ.

[19] Creswell JW (2011). Research Design: Qualitative, Quantitative, and Mixed Methods Approaches.

[20] Linnan L, Steckler A (2002). Process Evaluation for Public Health Interventions and Research.

[21] Braun V, Clarke V (2006). Using thematic analysis in psychology. Qual Res Psychol.

[22] Martin K, Mackworth-Young CRS, Nyamwanza R (2025). Financial incentives to improve uptake of partner treatment for sexually transmitted infections in antenatal care: a cluster randomised trial in Zimbabwe. Lancet Glob Health.

[23] Pinnock H, Barwick M, Carpenter CR (2017). Standards for Reporting Implementation Studies (StaRI) Statement. BMJ.

[24] O’Brien BC, Harris IB, Beckman TJ (2014). Standards for reporting qualitative research: a synthesis of recommendations. Acad Med.

[25] Joseph Davey DL, Shull HI, Billings JD (2016). Prevalence of Curable Sexually Transmitted Infections in Pregnant Women in Low- and Middle-Income Countries From 2010 to 2015: A Systematic Review. Sex Transm Dis.

[26] Nyemba DC, Medina-Marino A, Peters RPH (2021). Prevalence, incidence and associated risk factors of STIs during pregnancy in South Africa. Sex Transm Infect.

[27] Chaponda EB, Chico RM, Bruce J (2016). Malarial Infection and Curable Sexually Transmitted and Reproductive Tract Infections Among Pregnant Women in a Rural District of Zambia. Am J Trop Med Hyg.

[28] Shannon CL, Bristow C, Hoff N (2018). Acceptability and Feasibility of Rapid Chlamydial, Gonococcal, and Trichomonal Screening and Treatment in Pregnant Women in 6 Low- to Middle-Income Countries. Sex Transm Dis.

[29] Riddell MA, Vallely LM, Mengi A (2024). Point-of-care testing and treatment of sexually transmitted and genital infections to improve birth outcomes in high-burden, low-resource settings (WANTAIM): a pragmatic cluster randomised crossover trial in Papua New Guinea. Lancet Glob Health.

[30] Martin K, Dziva Chikwari C, Mackworth-Young CRS (2022). “It was difficult to offer same day results”: evaluation of community-based point-of-care testing for sexually transmitted infections among youth using the GeneXpert platform in Zimbabwe. BMC Health Serv Res.

[31] Duri K, Munjoma PT, Mataramvura H (2023). Antenatal hepatitis B virus sero-prevalence, risk factors, pregnancy outcomes and vertical transmission rate within 24 months after birth in a high HIV prevalence setting. BMC Infect Dis.

[32] Spearman CW, Andersson MI, Bright B (2023). A new approach to prevent, diagnose, and treat hepatitis B in Africa. *BMC Glob Public Health*.

[33] Zeng W, Lannes L, Mutasa R (2018). Utilization of Health Care and Burden of Out-of-Pocket Health Expenditure in Zimbabwe: Results from a National Household Survey. *Health Syst Reform*.

[34] (2025). Integrating point-of-care screening for curable sexually transmitted infections with hiv, syphilis and hepatitis b screening in antenatal care services in zimbabwe: a mixed-methods process evaluation lshtm data compass.

