# Supplementary figures and images for: Integrating point-of-care screening for curable sexually transmitted infections with HIV, syphilis and hepatitis B screening in antenatal care services in Zimbabwe: a mixed-methods process evaluation
Source: BMJ Glob Health. 2025 Dec 5;10(12):e019820. doi: 10.1136/bmjgh-2025-019820 (PMC12684121; doi:10.1136/bmjgh-2025-019820)

*Supplementary table A: Schematic diagram detailing order of daily study activities*


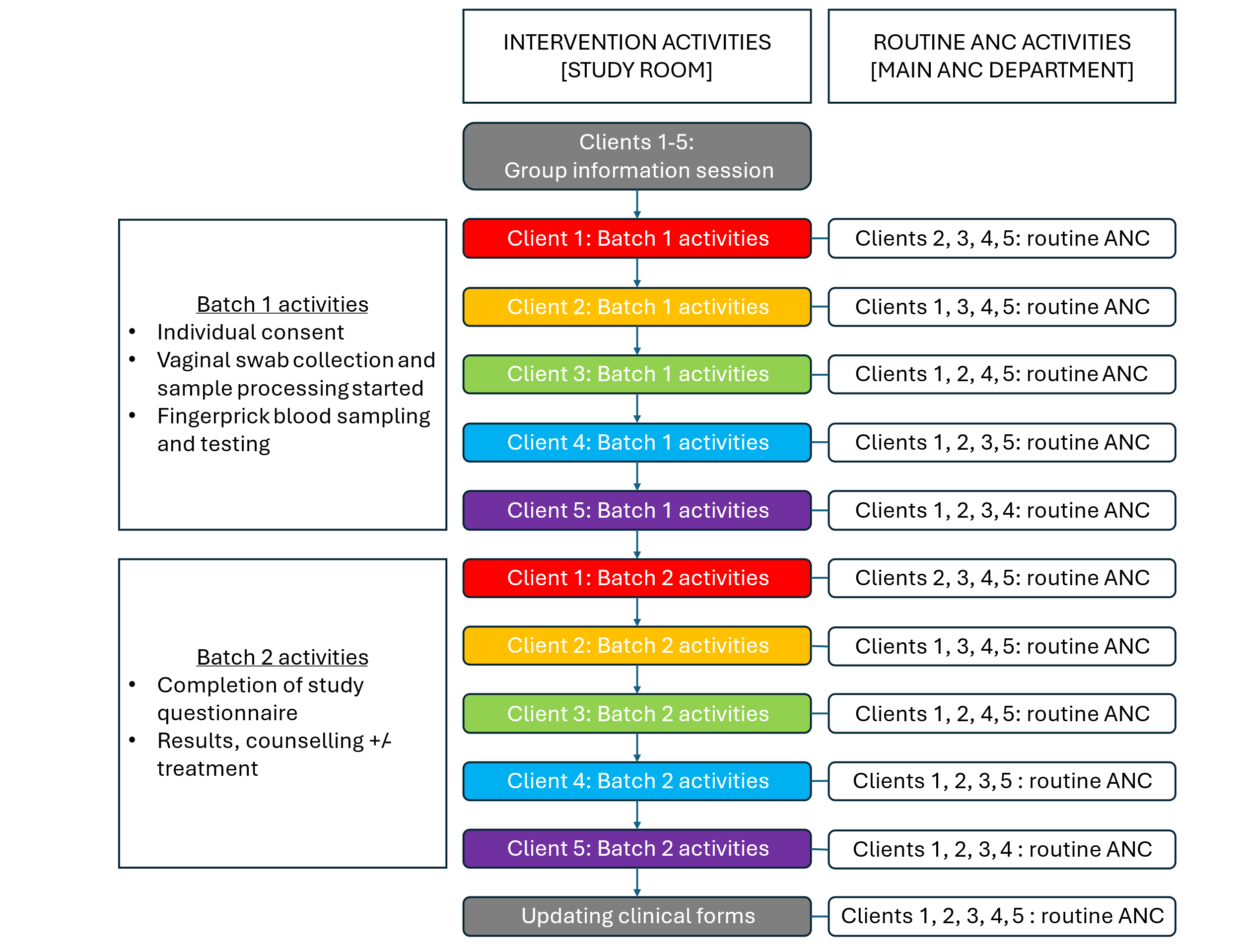

Supplement: online supplemental file 1 [file bmjgh-10-12-s001.docx]

Supplemental figure C: pathway to impact

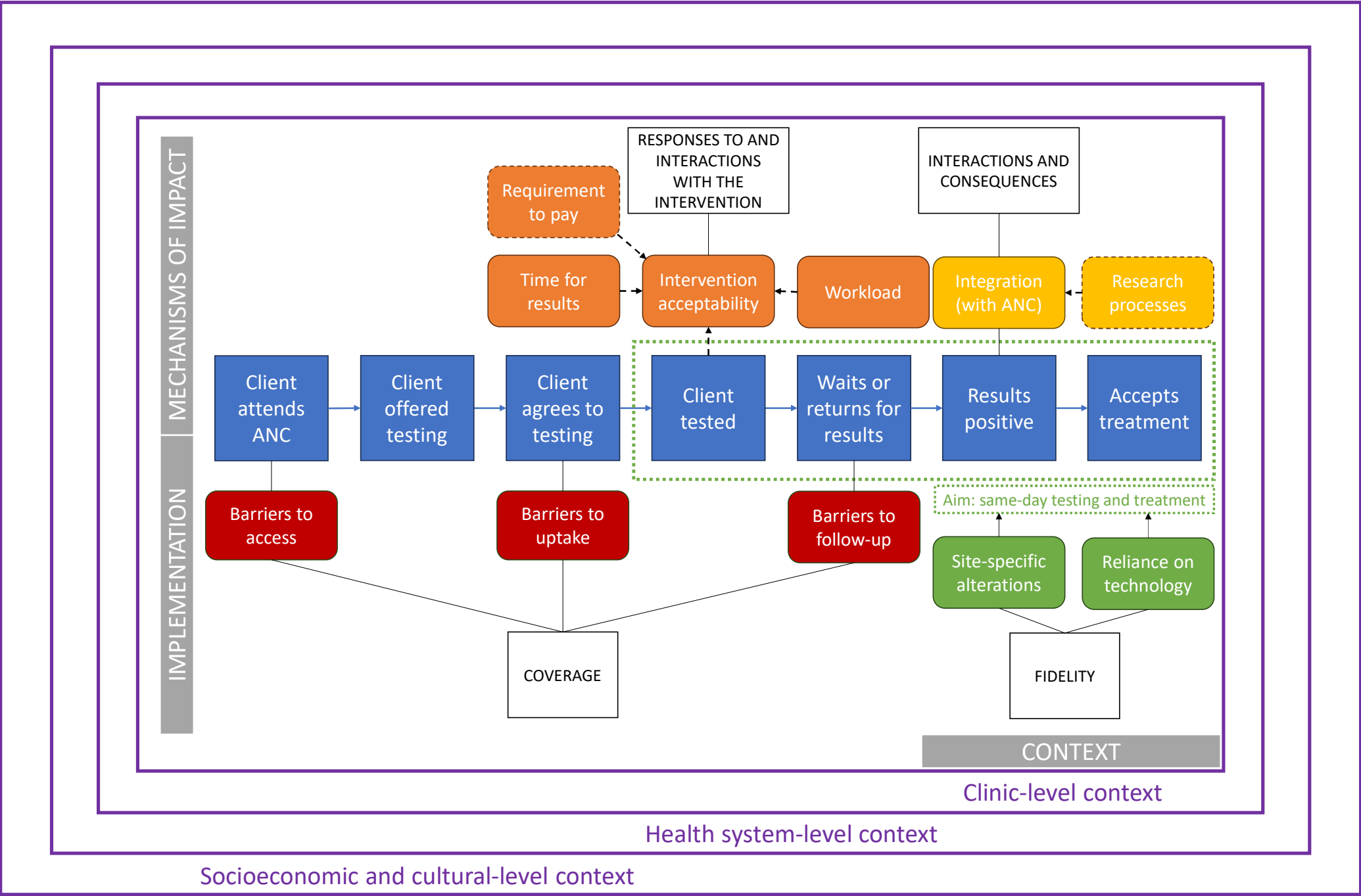

Supplement: online supplemental file 3 [file bmjgh-10-12-s003.pdf]
