# Supplementary material for: Integrating point-of-care screening for curable sexually transmitted infections with HIV, syphilis and hepatitis B screening in antenatal care services in Zimbabwe: a mixed-methods process evaluation
Source: BMJ Glob Health. 2025 Dec 5;10(12):e019820. doi: 10.1136/bmjgh-2025-019820 (PMC12684121; doi:10.1136/bmjgh-2025-019820)
Supplement: online supplemental file 2 [file bmjgh-10-12-s002.docx]

*Supplementary table B: Framework for process evaluation*

| Framework domain | Research domain | Research questions | Data collection methods and sources | Key results |
| --- | --- | --- | --- | --- |
| Implementation | Coverage | - What proportion of: (a) Pregnant women attending antenatal care were offered STI screening, (b) Pregnant women who were offered STI screening took it up (c) Positive STI cases were treated, (d) Partners of positive cases were treated? - How equitable was this coverage? - What were the barriers and facilitators to each step? | Intervention monitoring data  Structured observation  FGDs  IDIs | 8.2% (1105/13500) assessed for eligibility  99.8% (1103/1105) eligible  91.0% (1004/1103) accepted screening  99.7% (1000/1003) of those screened (with full set of results) collected results – 98.5% (988) on same day  100.0% (308/308) of curable STI cases treated – 98.4% (303) on same day  94.5% (291/308) of participants with a curable STI provided at least one partner slip  33.0% (96/291) partners treated  Factors influencing access, uptake and follow-up   - Access: ANC booking fees (US$25 – covers all ANC activities) - payment of clinic fees reported by 54.0% (540/1000), results-based financing vouchers facilitated access for those unable to afford clinic fees. Transport costs reported by 60.2% (601/999), - Uptake: Time constraints, not being comfortable with swab, stigma around screening - Follow-up: Large catchment area and ANC clients not residing locally |
|  | Fidelity | - How did implementation vary from the protocol i.e. (a) offering STI screening, (b) undertaking STI screening, (c) providing comprehensive case management including partner notification, (d) training and supervision of staff - What were the barriers and facilitators to implementation? - What adaptations were made and why? |  | - Testing offered on 94.7% (142/150) and 95.6% (65/68) of days where ANC was operating at sites A and B, respectively – not offered on 11 days due to intervention team leave. - Recruitment not possible on a 8 further days: national election period (n = 5); ANC staff training (n = 2), and ANC closure due to no water (n = 1) - For 988 participants who received same-day results, 97.4% (962/988) did not have to wait longer at the clinic for their results: site A = 98.1% (683/696); site B = 95.6% (279/292). - For participants who did have to wait longer at clinic for results: site A = median 60 minutes, IQR 55 – 75 minutes, range 20 to 120 minutes (n = 12); site B, median 30 minutes, IQR 30 – 60 minutes, range 10 – 120 minutes (n = 13) - Off-site testing performed at laboratory for 0.7% (7/1000) individuals due to a technical problem with GeneXpert laptop keyboard (n = 5), GeneXpert laptop battery running out (n = 1) and a GeneXpert error late in the day (n = 1). - GeneXpert errors affected 25 participants – for 24, a sample was re-run and a result produced. For the one individual, there was insufficient medium left in the swab container to re-run the sample and they did not want to provide another swab, so repeat test not performed. - 71.4% (10/14) of participants diagnosed with HBV successfully referred to hepatology clinic. - 66.7% (8/12) live birth newborns administered birth dose HBV vaccine - Reasons for non-administration of the birth dose vaccine included; participant or the father of the baby not consenting to vaccination (n=2); participant leaving Harare before birth with no plans to return (n=1); and administrative issues with one clinic requesting additional approvals for vaccine administration (n=1).   Reliance on technology   - Reliance on technology led to high potential for disruption. E.g. a malfunctioning laptop keyboard could have significantly derailed testing - keyboard replacement took several months, during which time there fortunately was access to a spare laptop with GeneXpert software installed. - Similarly, one module of the GeneXpert machine at site B started to have an increased error rate. As ANC processes proceeded more quickly at site B, machines at both sites were swapped with each other. - The capacity of the 4-module GeneXpert machine was also considered a limitation to scalability, with one intervention team member suggesting that laboratory testing would be required to scale up further.   Site-specific alterations to implementation   - Site A – study room hidden from main ANC waiting room, so keeping track of clients more challenging; higher client numbers; and ANC booking took whole day. - Site B – smaller number of clients allowed all clients to complete ANC procedures in tandem as a group, with all procedures complete by early afternoon. As a result, intervention team under additional pressure as integration of STI testing had to be completed within shorter time period. |
| Mechanisms of Impact | Responses to and interactions with the intervention | - Which components of the intervention were best accepted and adopted by pregnant women and HCWs and why? - What challenges and barriers were faced? | Structured Observation  FGDs  IDIs | Intervention acceptability   - High acceptability of intervention overall and for individual components - Acceptability enhanced by curability of majority of infections - For participants diagnosed with HBV, secondary care appointment and HBV birth dose vaccination also reported to be acceptable.   Barriers and challenges   - High workload for implementing team - The collection and testing of multiple sample types with different tests, with a range of reading times, was challenging - Small number of minor complaints about waiting time - Screening was provided free-of-charge within the study, and HBV clinic appointment fees were covered - many participants reported that they would not have been able to afford to pay for these, if required. - HBV contact tracing not completed satisfactorily |
|  | Interactions and Consequences | - How did various components of the intervention interact? - Were there any unanticipated pathways or consequences? |  | Integration   - Most components conducted by a dedicated team - Intervention reported to have integrated well with clinic processes where required - Challenges to integration included high clinic staff turnover and a heavy reliance on locum staff - Some reports of disruption to normal ANC processes - Research processes (e.g. written informed consent, detailed study questionnaire) were a challenge to integration, with reduced time available for intervention procedures. |
| Context | Proximal and distal | - What social, cultural, political, and logistical factors impede or facilitate how the intervention was implemented, and how were HCWs able to engage with and adopt aspects of the intervention? - What were contextual reasons for adaptations to the intervention and its delivery? | FGDs  IDIs  Structured and unstructured observations  Context diaries to record external events | Socioeconomic   - Volatile economic environment with significant infrastructural challenges and high levels of unemployment - Vulnerable youth with adolescent pregnancies and power imbalanced relationships - Social issues reported in communities: drug and alcohol abuse, sex work, high risk sexual behaviours. - Social isolation and lack of support networks resulted in intervention team taking on additional supportive and counselling roles.   Cultural-level   - Stigma associated with STIs and HIV, with strong association with promiscuity, as well as adolescent pregnancy. - Use of traditional and herbal medicines commonly reported - Strong patriarchal norms: household decisions are often male decisions. - Acceptance of male infidelity in relationships with onus placed on women to prevent this.   Health system-level   - Overburdened health system with many competing priorities - Poor basic clinic infrastructure, stockouts and out-of-pocket expenditure - High levels of staff attrition and reliance on locum staff   Clinic-level   - Unreliable water and electricity supply - Limited space at clinic led to inefficiencies in intervention - Poor mobile network at site A - Concerns about security so GeneXpert machine, laptop and powerpack transported daily to the clinic from the office |
