# Supplementary material for: Integrating point-of-care screening for curable sexually transmitted infections with HIV, syphilis and hepatitis B screening in antenatal care services in Zimbabwe: a mixed-methods process evaluation
Source: BMJ Glob Health. 2025 Dec 5;10(12):e019820. doi: 10.1136/bmjgh-2025-019820 (PMC12684121; doi:10.1136/bmjgh-2025-019820)
Supplement: online supplemental file 4 [file bmjgh-10-12-s004.docx]

*Supplementary table D: Themes and selected supporting quotes*

| Themes | Supporting quotes |
| --- | --- |
| Implementation | |
| Coverage | Barriers to access  “Lack of funds like not having money to come to the clinic and use to buy the needed consultation fees and for medication. That can hinder us pregnant mothers to come to the clinic but the will to come to the clinic will be there” (ANC client, FGD)  “Some of the clients say that they may get a dollar a day or even 2 dollars so, that is not much because it is the same money has to buy tomatoes still and then you will really see that the client coming to book for antenatal care they even come when they are very far along like maybe 8 and a half months pregnant. That will clearly show that the financial settings are not okay because they delayed coming to the clinic to book for antenatal care.” (RT)  Barriers to uptake  “I work in town in the CBD, and the schedule is hectic because of time and like now I wanted to liaise with the nurse on duty to give me first preference when the maternity books come so that I quickly go back to work.” (ANC client – declined enrolment in main study)  “I just want to say that you should take all pregnant mothers and not just choose 5 because there maybe others who are sitting in the queue who would want to get tested for the 5 additional tests but they feel embarrassed to be seen getting up.” (ANC client)  “Some clients also mentioned that they would rather not know about these STIs they don’t want to have high BP and affect or kill their unborn child.” (RT)  Barriers to follow-up  “It’s quite difficult considering [Site A], the location here in [Site A]. People who normally come here are not people who reside in [Site A] they come from [Site A], Waterfalls so sometimes we won’t have resources to do follow ups and sometimes even if we say a client must come to the clinic tomorrow; they may fail to come because they won’t have bus fare to come to the clinic and even I remember there was a client who came and got tested for syphilis; there were no injections at the clinic at the time and she was told to go and buy the injections at the pharmacy.” (testing counsellor)  “Sometimes we call like we try to call or even use our village health workers to do the follow-ups for us and if the client is not in the vicinity of [Site A] then there won’t be anything we can do. Our village health workers only do follow-ups with clients who live in the [Site A] area only. So, when we call at times the phone will be unreachable and at other times, we are given the wrong phone number and they even give wrong addresses so it is really quite difficult.” (testing counsellor) |
| Fidelity | Site specific alterations to implementation  “At [Site A] they had started already with IPSAZ and I guess the only limitation with [Site A] is the proximity of where they are doing their procedures like the ANC reception was hidden and you would have to be present seeing what’s going on. Unlike here it is an open space when one client is done you know that the next person is following otherwise other processes are all the same.” (RT)  “Hmmm, but like at [Site B] the clients who come were actually fewer than here at [Site A] because some clients are even sent back home. Usually, people who come here at [Site A] polyclinic will be from around [Site A], Epworth, Waterfalls Retreat. So, just imagine how many people will be there. Usually, the nurses would say they are taking about 42 for the day so, this means the rest would have to wake up early the next day to be among the number of people being taken on the day. But for [Site B], this was different as a few people would be seen. Around 12 or 12.30 the clients would be dismissed and gone through all the ANC booking processes. For [Site A], the processes are done slowly and then even break for lunch then continue thereafter and even for health talk I remember most of the times they would do it around 4pm when everyone has been seen and payments made.” (RT)  Reliance on technology  “The one [GeneXpert machine] that we started with had no issues at all, we then had challenges with the one we were given which was being used at [Site B] site. That one would abort samples right in the middle of running them; so at times we would rerun the specimen or send it to the office so that the client would get their results the next day but this was towards the end of our recruitment.” (RT)  “When they come for check-ups, they know they will be coming for quick things only; so, for us to keep them for a long time too it becomes a big setback for them. So, I am just imagining that taking a lot of people unless there are going to be many GeneXpert machines to use then maybe it might be manageable because the machine only takes 4 and one takes about 1 hour 30 minutes to finish processing… So, if the clients to be served become many the chances of having many errors will be very high and it might become a disaster.” (RT) |
| Mechanisms of impact | |
| Responses to and interactions with the intervention | Intervention acceptability  “Haa no, because being told that you have an STI whilst you are able to walk on your own and take treatment is better than to seek treatment when you are bedridden. I loved the IPSAZ program and I pray that they don’t end their program soon." (ANC client)  “Haa (laughs) I didn’t really feel moved by the results because the nurse told me that it is treatable. So, I figured that if I take my pills, I will be fine.” (ANC client)  “Hmmm, I think on choosing 5 you should increase the number because for a batch of 40 people it means 35 are going back home not knowing that they have some STI. So, I think you should test everyone who comes for antenatal booking. Because if I hadn’t been chosen on that very day, I wouldn’t know that I have hepatitis B.” (ANC client – HBV positive)  “This was a good experience for me because I wanted to know the results so that I protect my baby in the process. My husband works out of town so, I don’t really know what he will be up to when I am not there all the time.” (ANC client, FGD)  “Everything went well and even when the nurse was taking the samples, I felt comfortable. I thought that maybe the sample procedure would be painful but it wasn’t at all. I have no complaints at all.” (ANC client)  “I just want to thank the team for offering us this free testing strategy and even the way that the research team handles us we even created a short-term relationship and were free to talk about our family issues to them during the procedures. So, thank you very much for this opportunity and it shouldn’t stop here.” (ANC client, FGD)  “I didn’t feel hurt as much in fact I was happy because the help that I was going to get I would get it there. The team even said that any questions that I have, I can ask the specialist at Parirenyatwa he will answer me in full. The help that I will get will be protecting the child as well and I can live longer. So, that counselling helped me understand hence not becoming scared that much.” (ANC client – HBV positive)  Barriers and challenges: workload  “And also, there is a challenge that when we select the 5 clients to come to room 4; they are also needed in the ANC unit so at times when the work vehicle comes to pick us up, we won’t be done with giving our 5 clients treatments and results. It will really be hectic. I personally feel that the 5 clients are too much for one day so at times there is really too little time to cover everyone.” (RT)  “I think on the side of these ladies who are doing the research there will be a lot of workload and now it depends on whether you guys will have the appropriate resources to meet up the demand but to be honest it will be too much workload. Maybe there will be need to employ more people to assist the clients and covering more work.” (testing counsellor)  Barriers and challenges: time for results  “They came out after 1 hour 30 minutes. Well, for me I thought that this was too long because for someone to wait this long some people are not patient enough to wait for that. I think a second machine which is actually faster that 1 hour 30 minutes will be ideal.” (ANC client, FGD)  “No, I didn’t face any challenges but just that it is boring just to sit idle waiting for your turn.” (ANC client)  Barriers and challenges: payment  “Free tests are good because they make us be catered for. For someone like me I don’t want to lie right now things are not good financially; so, for me to say that I will be able to afford to pay haa I will be lying.” (ANC client – HBV positive)  “Maybe it would take me some time to gather the money for the payment because I am not working right now and the moment that I am going to consult with my relatives asking for assistance and them getting back to me with response that they will help me raise the fees haaaa it would really take some time I don’t want to lie.” (ANC client – HBV positive)  “Hmm, to be honest I don’t think I would be able to afford anything right now. Because even transport money to come to the clinic I borrowed so haa for tests I wouldn’t get it at all. So, this free of charge works for me.” (ANC client) |
| Interactions and consequences | Integration  “It does affect the workload even for the nurses but now I think they have seen that their clients are also being helped like when we treat their clients that will be less work on their part; they won’t have much to do. So, to them yes it distracts their flow and they get to come here looking for their clients but then we are working together. Like the time that we take some of the clients to come here the client is supposed to go for examination and they will miss that; but now they understand that it is good for them. The client will just join the queue from where they left off and they go back to tell the nurses that they were in room 4 and continue with the process.” (RT)  “To be honest, I didn’t really notice. When I came for ANC checkup, I knew that I would be spending the whole day here at the clinic. So, I didn’t really feel bored that the process was taking longer or miss out on other tests. When the samples were taken, I went back to the reception area to finish up the other processes so, I didn’t really observe that the waiting time was longer because I was busy with other processes as well.” (ANC client – HBV positive)  “It is really a complicated process and usually when shifts change that’s when the flow gets disrupted again with the nurses on the shift having to be informed first because when other clients come to our room, they won’t understand what is happening and that may make them even scold the pregnant mothers thinking that they were just loitering about.” (RT)  “Because at the ANC we book a lot of pregnant mothers so there is lot of documentation that we do. It doesn’t necessarily affect but if the chosen pregnant mothers get delayed too much in room 4 that’s when we decide to conduct health educational sessions without them. But I think it is a very good program.” (midwife)  “Yes, for the IPSAZ team you are taking too long and when you bring back your 5 clients maybe the bloods have been collected already haa that area I think you should fix that. You take too long with the patients which is something that I think can be reverted.” (midwife) |
| Context | |
| Socioeconomic | Adverse economic environment  “Some have boreholes so we often go there to fetch water. But during the dry season we face challenges because the water table will be below so we are then limited in terms of buckets that we should fetch. One family will be restricted to 2 buckets and the borehole is solar powered so, if it is cloudy the power won’t be able to pump the water enough. For those people who have larger families it becomes difficult because those 2 buckets are not enough to cater for bathing, washing, and using in the toilet. The other advantage is that we have [river name] river close by, so when push comes to shove people resort to going there to do their laundry but the water is not clean because it is infused with the sewage drainage as well. All those problems can cause health issues like cholera you know, so I believe that this is one major problem.” (ANC client – HBV positive)  “I can just say what made it difficult for me to get to the clinic on time was the issue of money. I didn’t have money to come for check-up and for this visit I had to borrow money from my sister and it becomes embarrassing for a grown-up woman like me having to ask for money for basic things from siblings yet they have their own lives to live.” (ANC client – HBV positive)  “He left [his job] in February this year. So, he is not going to work. He does some hustles here and there but right now we can safely say we are under his parents’ hands financially.” (ANC client)  Vulnerability of youth  “There is this particular case wherein a client was dumped by the guy and the guy even changed his phone number. At home, the girl’s parents had told her to go back to the boyfriend’s house yet the boyfriend changed even where he used to stay. The girl ended up sleeping outside for quite some days because of that. So, it was really too much and on top of that she had an STI. So, in such cases you can’t let someone like that just go home you need to counsel them like talking to them in-depth so that they don’t go back home and have suicidal thoughts or something like that. Especially when you see the state that they are in, the clients will be crying so it won’t be professional for us to just leave a client like that to go back home.” (RT)  “We actually have a client who we had to refer I think she was 15 years old and she fell pregnant unplanned so, the boy ran away. She went back home and the parents also chased her away and told her to go back where she got her pregnancy from and she had nowhere to go. She looked for her mothers’ relatives and the uncle welcomed her and gave her strict laws that she would eat whatever was available. So, she came here for booking and then tested positive to an STI; we treated her and that’s when she started narrating her story and then fortunately here at [Site A] there is MSF Adolescents clinic and she went there after getting treatment from us. At the adolescents’ clinic she was also referred to Musasa project where she got help as well and well-wishers who donated some baby clothes and she delivered her baby. Still the parents didn’t take her in, she came back and asked us if we could help her with a job so that she would take care of her baby.” (RT)  “This was really emotional for me and also for children who are heads of households they also come for testing and they are double orphans who end up living hungry and not having a proper place to stay.” (testing counsellor)  Social issues within communities  “Right, drug abuse in [Site A] is on the rise like cocaine, alcohol, and other types and because these people then will say they don’t work in the first place and they don’t want to fend for themselves and for those who are fending for themselves in the flea markets they end up engaging in risky behaviours and getting the drugs. So, eventually they are into prostitution, selling the illegal things and that will increase the unsafe activities.” (nurse in charge)  “Yes, brothels are everywhere these days even here in [Site A] they are all over especially that part of flats there it is a base; if we go to the Beatrice cottages the area is infested with brothels and as long as one decided to engage in extra-marital affairs STIs are bound to be spread.” (partner)  “The problems in the community are the diseases that are infecting people especially in National, [Site A] hmmm a lot of promiscuous activities are happening. People are infecting each other with HIV then like in the locations if a cholera outbreak starts, that’s where it usually records a lot of deaths. Those are the things that are affecting us, but mostly the STIs haa a lot of people are coming to the clinic for treatment. People are engaging in too much risky behaviours.” (ANC client)  “To be honest I spend most of my time at work and when I am at home; I will be busy doing thorough cleaning and laundry. I don’t really mingle with other people in my community.” (ANC client – declined enrolment in main study) |
| Cultural | Stigma  “I know that STIs are sexually transmitted diseases that come through having multiple sexual partners let’s say if you are a woman and you have multiple sexual partners you will end up contracting these STIs.” (ANC client)  “Aaah I think the experience of coming to get tested is a good one you know because you might be relaxed at home thinking that you are still fit yet you are rotten already with HIV or other STIs. So, coming to get tested is the best thing that one can do.” (ANC client)  “I don’t think I am going to tell anyone. Maybe I will just die without telling anyone.” (ANC client)  “Well, for me I think at times we should have a separate room away from the other rooms which is private because when we got into room 4 and out people would know that this is the STI testing room. People won’t know that the STI tests were performed on anyone who wanted and not people who knew that they had STIs already. The room was at an open space like when you came out you would see the other clients at the reception. So, I think if it is a room which is away from the ANC building that will do. You will get in and no one will even know what you were doing in that room.” (ANC client, FGD)  “Those cases are quite common and we offer counselling to them like letting them know that they have to be there for their child because some men leave forever. So, the single mother or the unmarried mother has to be disciplined, having one child out of wedlock doesn’t mean that they have to go around sleeping with other men at the end of the day having another child again. They should learn from their mistakes and move on.” (testing counsellor)  Faith and tradition  “Maybe I still remember from school, but at the clinic I want to be honest I have never gone there to get information. Like for my case, since I have come here for treatment, I am also supposed to confess at church that I took medicines which we are not supposed to take. It should be natural remedies all the way. So, when my wife also came and told me that I am needed at the clinic I just told myself that I shouldn’t argue but comply and get the treatment since I had been experiencing some infection problems.” (partner)  “I think even giving health education to the pregnant mothers especially on the dangers of not treating syphilis because some of these people are not aware that one will not be feeling anything physically to show that they are sick so, when they come here the syphilis test comes out positive and they start thinking that maybe it is evil spirits causing that since they are pregnant. So, that health talk is important like properly telling them what syphilis is all about.” (testing counsellor)  “Haa, usually when I come here, I just hear a lot of women talking about traditional medicine to drink to pave way for the baby before being born. Different women say different remedies that they use and so, I will be listening to the different opinions that’s all.” (ANC client)  Patriarchy and gender roles  “These men will really humble you; you will be at home playing with kids and being the faithful housewife yet your husband is out there having sex with another person or other extra marital affairs.” (ANC client)  “I wanted to find out my status in terms of the STIs and since I don’t trust my husband that much.” (ANC client, FGD)  “And if you encourage the women to use condoms until the partner comes for treatment that will cause havoc in the marriage as the partner will refuse to use the condoms.” (testing counsellor)  “Aaah, I think we may have an increased chance and it now differs with the husbands that we have. For example, I am pregnant now; I may fail to give my husband his conjugal rights when he wants through the condition that I am in. But we are always encouraged to give him sex, because if we don’t do that; the husband will have an extra marital affair and that increases my chances of getting infected with the STI. So, we are always encouraged to give our husbands sex in different styles since we will be pregnant it might become difficult for him to get on top of me so that they don’t find an extra marital affair to have sex with.” (ANC client)  “We always tell the pregnant mothers that when they are pregnant they shouldn’t deny their husbands of sex and they have that belief that when you are heavily pregnant you shouldn’t sleep with your husband. That’s when the husbands start having extra-marital affair and that’s how the STIs come about… We always encourage the pregnant mothers to give their husbands the full conjugal rights from conception till they give birth so that they avoid the start of extra marital affairs and small houses. That way the levels of STIs may be reduced.” (nurse aid) |
| Health system | Overburdened and underfunded healthcare system  “I also think that the syphilis medication must be always available because one thing that makes some clients not come back is the issue of being instructed to buy medication from outside or the pharmacy because the clinic doesn’t have the medication. Most clients cannot afford to buy the medication and they don’t come back for treatment. So, I think if the medication is available at the clinic it might help.” (testing counsellor)  “So, those are some of the challenges and also, we don’t have adequate medicine to give to the patients. If we write prescriptions we don’t know if the patient is really going to buy the medicine at the pharmacy or not and whether they have the money or not. Those are the difficulties.” (midwife)  “If the patient is transferred it is a plus on the bill. The USD25 that they pay as booking fee doesn’t cover ambulance fee. Ambulance fee is paid separately. Most of the clients won’t have money to cater for the ambulance fee.” (nurse in charge)  Staff shortages and brain drain  “There was only one time that we came and we had BP and temperature taken only and the reason for that was because there was a shortage of nurses on that day… They just said that they experienced a challenge of having one nurse at the ANC station; so probably with the number of pregnant women that had come on that day the workload was too much so BP, and temperature were taken and if it was normal then we were dismissed. Those who had abnormal temperature were told to remain behind. On the next visit, everything was back to normal and we were checked for everything.” (ANC client – HBV positive)  “I think staff should be increased because the waiting period of clients is the only factor that clients complain about. Like per day we are seeing 70 clients and we have only 2 PCCs testing these clients. So, the waiting period will really be too much for the clients. Because one client might test positive to HIV and she needs time for counselling and accept the new status and hence be started on ART. So, we really need to improve on the service providers like increasing the number of staff.” (midwife)  “We would do the processes that would make us get participants. When we got here every morning, we would make sure that we talked to the nurses on duty on the day because here at [Site A] they have several locum nurses. So, you would find that the next day that you come Sister XXXXX won’t be the one responsible for the ANC checkups. So, we had to thoroughly explain our study to the new faces almost every day so that they know that we take 5 clients from their pool and they have to come back to them as well for other processes.” (RT) |
| Clinic-level | “Yes, the water supply would be disrupted; often the water would not come out of the taps but we had our own backup of those 5litre bottles that we stocked on the sink to ensure that every time we needed to use the toilet, we would have water to flush. So, we sorted ourselves there. At times when the water wasn’t there for some days the sister-in-charge would tell us that they are not taking any clients for the day.” (RT)  “Electricity is always coming and going like at times it would come back in the middle of work and all but fortunately for us we had a backup battery that we used so, it didn’t really affect our work. Our machines would always be charged and hence not affecting the work that we did. But when we used the battery, it meant that we had to recharge it at the office for tomorrow in case there is no power again and that meant that we had to come back with the powerpack to the office after knocking off at the clinic.” (RT)  “I think having a bigger room unlike the one which is there. A bigger room will enable clients to move around freely because the one is smaller, I remember when we did group consenting process, we sat on the beds for us to fit in as 5 clients.” (ANC client, FGD) |

ANC = Antenatal care. FGD = Focus group discussion. HBV – Hepatitis B virus. RT = Research team.
