# Supplementary material for: Integrating point-of-care screening for curable sexually transmitted infections with HIV, syphilis and hepatitis B screening in antenatal care services in Zimbabwe: a mixed-methods process evaluation
Source: BMJ Glob Health. 2025 Dec 5;10(12):e019820. doi: 10.1136/bmjgh-2025-019820 (PMC12684121; doi:10.1136/bmjgh-2025-019820)
Supplement: online supplemental file 5 [file bmjgh-10-12-s005.docx]

*Supplementary material E: Reflexivity statement*

1. **How does this study address local research and policy priorities?**

Colleagues from the AIDS and TB unit of the Zimbabwe Ministry of Health and Child Care (MOHCC) were involved at the study proposal and funding application stage to ensure that the study aligned with local policy priorities. Management of sexually transmitted infections (STIs) in Zimbabwe relies on syndromic management. In addition to not allowing for the detection and treatment of asymptomatic STIs, it also means that aetiological surveillance of STIs is lacking, which is important to guide the development of guidelines. There is no recent data available on STI prevalence in antenatal care in Zimbabwe. To our knowledge, this is the largest study investigating prevalence of *Chlamydia trachomatis*, *Neisseria gonorrhoeae* and *Trichomonas vaginalis* in antenatal care in Zimbabwe, and one of the largest for hepatitis B (HBV). The aetiological data produced in this study can be used to inform future development of guidelines in Zimbabwe. Furthermore, the quantitative and qualitative data provides important lessons for implementation of screening for both STIs and HBV in a Zimbabwean context. In particular, HBV screening as part of the triple elimination initiative is currently under consideration by MOHCC, and so findings will be directly relevant to such a rollout. Our findings has also directly informed a funded cluster randomised trial in Zimbabwe (PROMISE) investigating where antenatal STI screening can prevent adverse birth outcomes. Implementation of gonococcal culture and drug sensitivity testing as part of the broader project also informed the development of the Zimbabwe county-specific protocol for the ongoing enhanced gonococcal antimicrobial surveillance programme.

1. **How were local researchers involved in study design?**

This study is the primary output of a PhD fellowship in global health research held by KM, a researcher from a high-income setting. As such, design of the study was led by KM. However, local researchers, clinicians, and colleagues from the MOHCC were involved in study design at the proposal stage and throughout. Specifically, ED and TB were involved in design of study administration and data management processes, respectively. CDC, FN, OM and AM provided guidance with regards to implementation of STI screening within the Zimbabwean health system. OM and AM provided guidance to ensure the project aligned with MOHCC priorities. LK provided input with regards to HBV screening and the development of a referral pathway to secondary care, for individuals diagnosed with HBV.

1. **How has funding been used to support the local research team(s)?**

RN and the intervention delivery team were directly funded through the project. Additionally, the project was used as a platform for other sub-studies (not presented in this manuscript) aimed at the development of a mental health intervention for pregnant women and a cohort study focussed on pregnant children (the “kids with kids” study).

1. **How are research staff who conducted data collection acknowledged?**

Staff involved in collection of qualitative and process data through observations, interviews and group discussions (RN, MT) are included as authors in the manuscript. Staff involved with delivery of the intervention are included in the acknowledgements section.

1. **How have members of the research partnership been provided with access to study data?**

Members of the Biomedical Research and Training Institute (BRTI) data team have direct access to the raw data on ODK servers hosted by the London School of Hygiene & Tropical Medicine. All members of the partnership have access to the study data.

1. **How were data used to develop analytical skills within the partnership?**

This study presents the main output of a PhD fellowship for KM. As a result, analysis was primarily conducted by KM. However, the “Kids with kids” sub-study allowed for a junior social scientist to further develop qualitative data collection and analysis skills.

1. **How have research partners collaborated in interpreting study data?**

Interim study results were presented at an STI advisory meeting hosted by the MOHCC and attended by both local and international stakeholders, as well as at an annual research day hosted by the BRTI. Both events allowed research partners and colleagues to provide feedback and insight into ongoing research processes. All co-authors have been involved in interpretation of study findings.

1. **How were research partners supported to develop writing skills?**

This study presents the main output of a PhD fellowship for KM. As a result, KM wrote the first draft of the manuscript. All authors reviewed the final draft of the manuscript.

1. **How will research products be shared to address local needs?**

In addition to findings having been presented at the STI advisory meeting hosted by the MOHCC and the BRTI annual research day, KM also presented study findings as part of a “booth talk” at the “International Conference on AIDS and STIs in Africa 2023, held in Harare, Zimbabwe. Findings have also been presented directly to the MOHCC, and further dissemination is expected at early career researcher meetings hosted by a collaboration between BRTI and the University of Zimbabwe.

1. **How is the leadership, contribution and ownership of this work by LMIC researchers recognised within the authorship?**

The leadership, contribution, and ownership of this work by LMIC researchers is acknowledged through the nine co-authors who are LMIC researchers, each of whom provided critical input into study design and interpretation.

1. **How have early career researchers across the partnership been included within the authorship team?**

The first author (KM) is an early career researcher completing a doctoral fellowship, for which this is the primary output. Additionally, CRSMY, RN, CDC and MT are all early career researchers.

1. **How has gender balance been addressed within the authorship?**

Ten authors are female and seven authors are male.

1. **How has the project contributed to training of LMIC researchers?**

Training was provided in qualitative and quantitative data collection, as well as clinical procedures including the use of novel diagnostics.

1. **How has the project contributed to improvements in local infrastructure?**

This project has not directly contributed to improvements in local infrastructure.

1. **What safeguarding procedures were used to protect local study participants and researchers?**

All data collected was anonymised using a unique study ID. Any identifiable data (e.g. locator forms) were stored in secure, locked facilities with access limited to the study team. For vulnerable participants, the intervention team worked with clinic staff and NGO partners to support and refer them on if necessary, using existing pathways.
